# Biomarker testing patterns among patients newly diagnosed with metastatic non-small cell lung cancer, prostate cancer, and bladder cancer

**DOI:** 10.1093/oncolo/oyag243

**Published:** 2026-06-27

**Authors:** Siraje Mahmud, Lisa Dwyer Orr, Mika Cline, Ayda Mirsalehi, Monica Lisi, Laura Ensley, Lorraine Brisbin, R Steven Paulson

**Affiliations:** Precision Health Informatics, LLC, Dallas, TX 75251, United States; Johnson & Johnson, Titusville, NJ 08560, United States; Texas Oncology, Dallas, TX 75251, United States; Precision Health Informatics, LLC, Dallas, TX 75251, United States; Precision Health Informatics, LLC, Dallas, TX 75251, United States; Precision Health Informatics, LLC, Dallas, TX 75251, United States; Precision Health Informatics, LLC, Dallas, TX 75251, United States; Texas Oncology, Dallas, TX 75251, United States

**Keywords:** non-small cell lung cancer, prostate cancer, bladder cancer, biomarker, molecular analysis

## Abstract

**Background:**

National clinical guidelines recommend biomarker testing to identify actionable alterations across cancers, supporting personalized treatment.

**Methods:**

This retrospective database study characterized biomarker testing patterns among adult patients newly diagnosed with metastatic non-small cell lung cancer (NSCLC), prostate cancer, or bladder cancer (January 2018–December 2022), receiving care within a large regional community oncology network in the West South-Central United States.

**Results:**

Of 18 491 patients with NSCLC, 20 810 with prostate cancer, and 4120 with bladder cancer, 7383 (40%), 4985 (24%), and 888 (22%), respectively, had metastatic disease. For these metastatic subgroups, biomarker testing rates were 80% (NSCLC), 34% (prostate), and 42% (bladder). Median time from disease diagnosis to first test order was 10 days (NSCLC), 98 days (prostate), and 23 days (bladder), with differences observed across racial and ethnic groups. Among tested patients, actionable mutations were identified in 29% (NSCLC), 25% (prostate), and 14% (bladder) of patients, and targeted therapy was received by 54%, 15%, and 38% of these patients, respectively. Among patients who initiated treatment prior to receipt of test results, 72% (NSCLC), 12% (prostate), and 30% (bladder) subsequently switched to targeted therapy following a positive result. Testing rates increased between 2018 and 2022 for all cohorts; result turnaround times improved in prostate and bladder cancers.

**Conclusions:**

Biomarker testing was highest in metastatic NSCLC and lower in prostate and bladder cancer in this community oncology setting. Despite improvements over time, gaps remain in timely testing and use of targeted therapies, highlighting opportunities to optimize precision oncology implementation.

Implications for Practice Molecular biomarker testing among patients newly diagnosed with metastatic non-small cell lung cancer has improved in community settings in the United States but remains lower and more variable for those newly diagnosed with prostate or bladder cancer. Realizing the benefits of targeted therapies depends on timely and appropriate testing. This study highlights the need to address both educational and system-level barriers to biomarker testing in community practice, including workflow, access, and turnaround time challenges. Efforts to standardize testing pathways and improve care coordination may help advance more equitable implementation of precision oncology.

## Introduction

Biomarker testing is central to precision oncology and enables identification of actionable molecular alterations that may inform prognosis and selection of targeted or immunotherapeutic treatment strategies. Real-world data show that biomarker testing is associated with reduced mortality risk in patients with metastatic cancer.^1^ National Comprehensive Cancer Network^®^ (NCCN^®^) Clinical Practice Guidelines in Oncology (NCCN Guidelines^®^) focus recommendations for molecular biomarker testing and targeted therapy on advanced or metastatic cancers with actionable biomarkers.^2–4^ Despite this guidance, studies have indicated that biomarker testing has been variable in the community setting in the United States.^5–7^

Approximately 85% of patients with cancer in the United States receive care in community oncology practices.^8^ Biomarker testing rates have increased over time;^9–11^ however, the evidence suggests that rates remain lower in community versus academic settings^9^^,^^12^^,^^13^ and turnaround times of results can be several weeks.^14^ Additionally, differences persist among minoritized populations and socioeconomically disadvantaged groups.^6^^,^^9^^,^^10^^,^^15^ Importantly, biomarker testing practices and clinical utility differ substantially across tumor types, reflecting differences in guideline recommendations, the breadth of actionable alterations, and the maturity of targeted treatment landscapes.

Lung, prostate, and bladder cancers are among the most common cancers in the United States.^16^^,^^17^ Combined, they account for 46% of cancer diagnoses in men and 14% in women.^16^^,^^17^ Approximately 60% of patients with advanced or metastatic non-small cell lung cancer (NSCLC) are estimated to have an actionable mutation.^18^^,^^19^ Potential actionable mutations in homologous recombination repair (HRR) genes are found in ∼20%-30% of advanced or metastatic prostate cancer.^20^^,^^21^ In bladder cancer, 70% and 15% of patients with non-muscle-invasive and muscle-invasive disease, respectively, are estimated to have *FGFR3* mutations/fusions.^22^ However, despite progress in the molecular characterization of these cancers and the advent of targeted therapies and immunotherapies,^23–25^ mortality rates remain high. Collectively, NSCLC, prostate cancer, and bladder cancer are estimated to account for nearly one-third of cancer-related deaths in the United States.^16^^,^^17^

This study aimed to characterize biomarker testing patterns and associated treatment use among patients diagnosed with metastatic NSCLC, prostate cancer, or bladder cancer within a large regional community oncology network in the West South-Central United States. Although prior studies have evaluated biomarker testing within individual tumor types, few real-world analyses have examined testing patterns and treatment integration across multiple cancers within the same clinical network. During the study period (2018-2022), biomarker testing recommendations and therapeutic options evolved substantially across tumor types, contributing to variability in testing patterns and treatment adoption in clinical practice; however, for the purposes of this analysis, biomarker actionability was defined using a consistent framework applied across the study period.

## Patients and methods

### Study design and data source

All data were de-identified in accordance with the Health Insurance Portability and Accountability Act (HIPAA). This retrospective database analysis included adults (aged ≥18 years) newly diagnosed with NSCLC, prostate cancer, or bladder cancer between January 1, 2018, and December 31, 2022. The patients received care from Texas Oncology, a large regional community oncology network in the West South-Central United States. Patients enrolled in a clinical trial were excluded.

Anonymized patient-level discrete structured and non-structured data and electronic medical record (EMR) abstraction came from three sources, together, covering ∼1.7 million distinct cancer patients: (1) the iKnowMed EMR database; (2) the ELLKAY CareEvolve database, a real-time genetic HL7-mapped structured database; and (3) a proprietary molecular data warehouse operated by Precision Health Informatics, LLC. Recent improvements to the EMR database system, HL7 integration of external databases, and use of natural language processing allowed for maximal capture of testing results. The Western Institutional Review Board, Inc. determined that the research was exempt from institutional review board oversight and approved waivers of informed consent and HIPAA authorization.

### Variables

Biomarker testing was defined based on tumor-specific guideline-relevant alterations (Table S1). Actionable alterations were defined according to guideline frameworks available at the time of analysis (ie, ver.1.2023) and were assessed among patients who underwent biomarker testing. Programmed death-ligand 1 expression and microsatellite instability/mismatch repair testing were not included in the definition of actionable biomarkers for this analysis. Clinical characteristics included patient demographics (ie, age, sex, race and ethnicity, and primary insurance status), clinical characteristics (ie, comorbidities, disease stage, and histology), biomarker test details (ie, sample origin, and type of test), time from diagnosis to first biomarker test order, and time from test order to receipt of test results. The date of diagnosis was defined as the earliest pathology-confirmed diagnosis date recorded in the EMR. Test order date was defined as laboratory receipt date. Proportions of actionable alterations were calculated among tested patients, and proportions of targeted therapy use were calculated among patients with identified actionable alterations.

### Statistical analysis

Descriptive statistics were used to summarize baseline demographics, clinical characteristics, and outcomes of interest. For time-to-event analyses, Kaplan–Meier (KM) methods were used to obtain medians and 95% confidence intervals (CIs). Patients were followed from their initial molecular test date until death, last activity in the database, or end of the follow-up period (December 31, 2022), whichever occurred earliest. Subgroup analyses assessed outcomes by baseline race and ethnicity. All analyses were performed using Excel and Python. All analyses were descriptive and exploratory in nature and are intended to be hypothesis-generating; no causal inferences were made.

## Results

### Study population

Data were collected for 43 719 patients newly diagnosed with NSCLC, prostate cancer, or bladder cancer in community practices between January 1, 2018, and December 31, 2022. Of these, 298 were enrolled in clinical trials or had possible small cell lung carcinoma and were excluded from the analyses.

**Figure 1.**
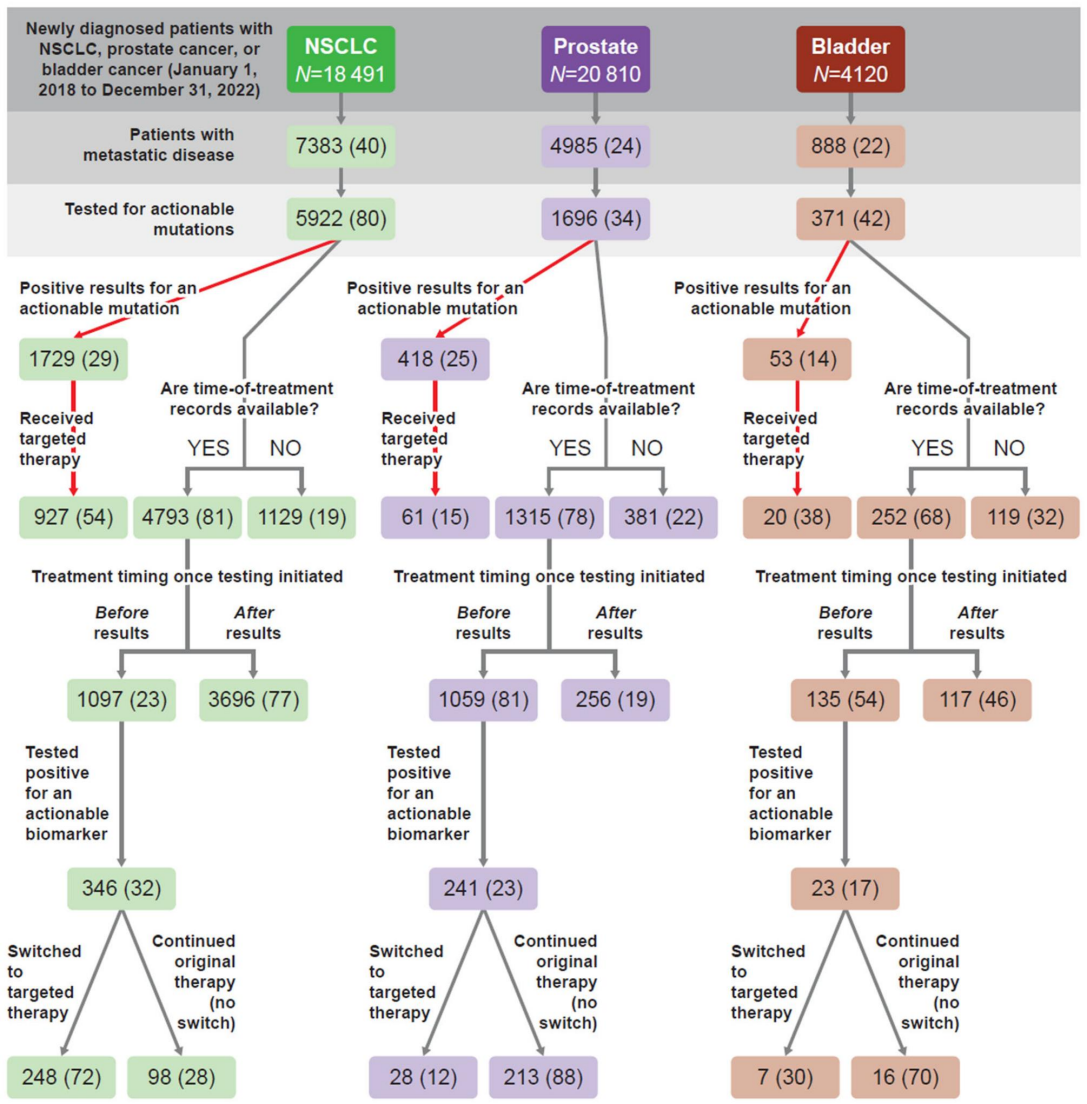
Flow diagram of patient records and testing results, organized by cancer type. Data are presented as *n* (%). NSCLC, non-small cell lung cancer.

**Figure 2.**
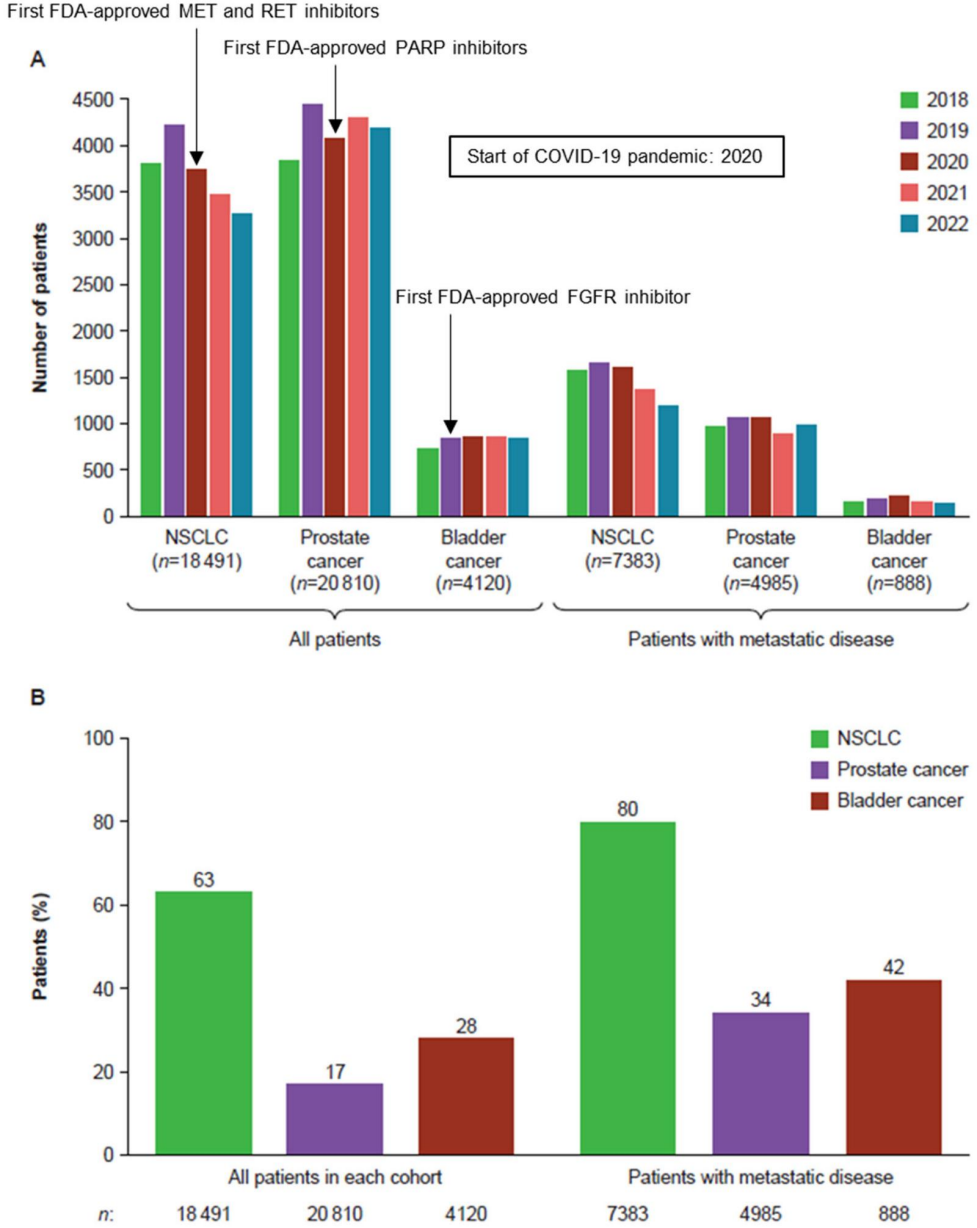
(A) Numbers of patients newly diagnosed with NSCLC, prostate cancer, or bladder cancer by year. (B) Percentages of all patients per cancer cohort and those with metastatic disease per cohort who received biomarker testing during the study period. FDA, US Food and Drug Administration; NSCLC, non-small cell lung cancer; PARP, poly(ADP-ribose) polymerase.

The final overall study population included 18 491 patients newly diagnosed with NSCLC, 20 810 with prostate cancer, and 4120 with bladder cancer (13% non-muscle invasive, 60% muscle invasive [including metastatic and non-metastatic disease], 27% unknown) (Figure 1). Of these, 7383 (40%) patients with NSCLC, 4985 (24%) with prostate cancer, and 888 (22%) with bladder cancer had metastatic disease at diagnosis. A decreasing trend in NSCLC diagnoses was observed beginning in 2020 and continuing through 2022 (Figure 2A), which may reflect disruptions in cancer detection during the COVID-19 pandemic. This reduction contrasted with prostate and bladder cancers, where the case numbers remained relatively stable.

Other than sex, patient demographics were broadly similar across the three cancer cohorts (Table 1). Mean age at time of disease diagnosis was 71.0-73.1 years. Males comprised 51% of the NSCLC cohort, 100% of the prostate cancer cohort, and 73% of the bladder cancer cohort.

**Table 1:** Patient demographics and clinical characteristics of the overall study population by cancer type.

Parameter	NSCLC	Prostate cancer	Bladder cancer
	(*N *= 18 491)	(*N *= 20 810)	(*N *= 4120)
**Age, years**			
**Mean ± SD**	71.0 ± 9.8	71.1 ± 9.1	73.1 ± 11.0
**Median (range)**	72 (19-100)	71 (22-102)	74 (20-104)
**Sex**			
**Female**	9106 (49)	0	1103 (27)
**Male**	9385 (51)	20 810 (100)	3017 (73)
**Race or ethnicity**			
**White**	9808 (53)	9517 (46)	2224 (54)
**Black/African American**	1007 (5)	1491 (7)	154 (4)
**Asian**	252 (1)	165 (1)	35 (1)
**Hispanic/Latino^a^**	1856 (10)	2663 (13)	458 (11)
**Other^b^**	3957 (21)	5366 (26)	926 (22)
**Unknown**	1611 (9)	1608 (8)	323 (8)
**Stage at diagnosis**			
**0**	25 (<1)	—	222 (5)
**I**	3715 (20)	1615 (8)	322 (8)
**II**	1551 (8)	5024 (24)	954 (23)
**III**	3951 (21)	2870 (14)	619 (15)
**IV**	7383 (40)	4985 (24)	888 (22)
**Unknown**	1866 (10)	6316 (30)	1115 (27)
**KPS/ECOG PS^c^**			
**KPS 90-100/ECOG PS 0**	6013 (33)	11 372 (55)	1610 (39)
**KPS 70-80/ECOG PS 1**	7265 (39)	3368 (16)	1079 (26)
**KPS 0-40/ECOG PS 2-5**	1344 (7)	565 (3)	196 (5)
**Unknown**	3869 (21)	5505 (26)	1235 (30)
**Histopathology**			
**Adenocarcinoma**	9821 (53)	2973 (14)	—
**Squamous cell carcinoma**	4726 (26)	2 (<1)	—
**Other**	220 (1)^d^	32 (<1)^e^	—
**MIBC**	—	—	2461 (60)
**NMIBC**	—	—	544 (13)
**Unknown**	3724 (20)	17 803 (86)	1115 (27)
**Gleason score**			
**2-5**	—	39 (<1)	—
**6**	—	1275 (6)	—
**7**	—	3744 (18)	—
**8-10**	—	2905 (14)	—
**Unknown**	—	12 847 (62)	—
**Comorbidities^f^**			
**Other cancer**	3110 (17)	2910 (14)	833 (20)
**Hypertension**	2001 (11)	2536 (12)	558 (14)
**Nutritional deficiency**	1292 (7)	1672 (8)	469 (11)
**Hyperlipidemia**	987 (5)	1376 (7)	292 (7)
**Diabetes**	804 (4)	1008 (5)	257 (6)
**Smoking history**			
**Current or former smoker**	8610 (47)	5434 (26)	1378 (33)
**Never smoked**	2413 (13)	6379 (31)	956 (23)
**Unknown**	7468 (40)	8997 (43)	1786 (43)
**Provider practice location**			
**Rural**	129 (<1)	105 (<1)	24 (<1)
**Urban**	18 362 (99)	20 705 (99)	4096 (99)
**Primary insurance type**			
**Commercial/Medicare Advantage**	9960 (54)	12 540 (60)	2300 (56)
**Medicare/Medicaid**	8418 (46)	8169 (39)	1791 (43)
**Unknown**	113 (<1)	101 (<1)	29 (<1)

Data are presented as *n* (%) unless otherwise stated.

Abbreviations: ECOG PS, Eastern Cooperative Oncology Group performance status; KPS, Karnofsky Performance Status; MIBC, muscle-invasive bladder cancer; NMIBC, non-muscle-invasive bladder cancer; NSCLC, non-small cell lung cancer; SD, standard deviation.

a Includes Hispanic/Latino, American Indian/Alaska Native, Hispanic Asian, Hispanic Black or African American, Hispanic multirace, Hispanic Native Hawaiian/Other Pacific Islander, Hispanic other, and Hispanic White (but excludes unknown/declined to inform).

b Non-Hispanic with multiple races and ethnicities chosen.

c ECOG PS scores were preferred over KPS where both were present.

d Large cell carcinoma (*n *= 214) and adenosquamous carcinoma (*n *= 6).

e Small cell carcinoma.

f Comorbidities occurring in ≥5% of patients in any cohort.

### Biomarker testing in metastatic disease

The proportion of patients with metastatic disease undergoing biomarker testing varied across tumor types. The highest testing rate was observed in patients with metastatic NSCLC (80% [5922/7383]), followed by metastatic bladder cancer (42% [371/888]) and metastatic prostate cancer (34% [1696/4985]) (Figures 1 and 2B). For the overall cohorts, the testing rates were 63% (11 577/18 491), 17% (3439/20 810), and 28% (1170/4120) for NSCLC, prostate cancer, and bladder cancer, respectively (Figure 2B). In addition, consistent with NCCN recommendations at the time of the analysis, the majority of tests were ordered as multigene panels rather than single-gene assays (Table S2).

**Figure 3.**
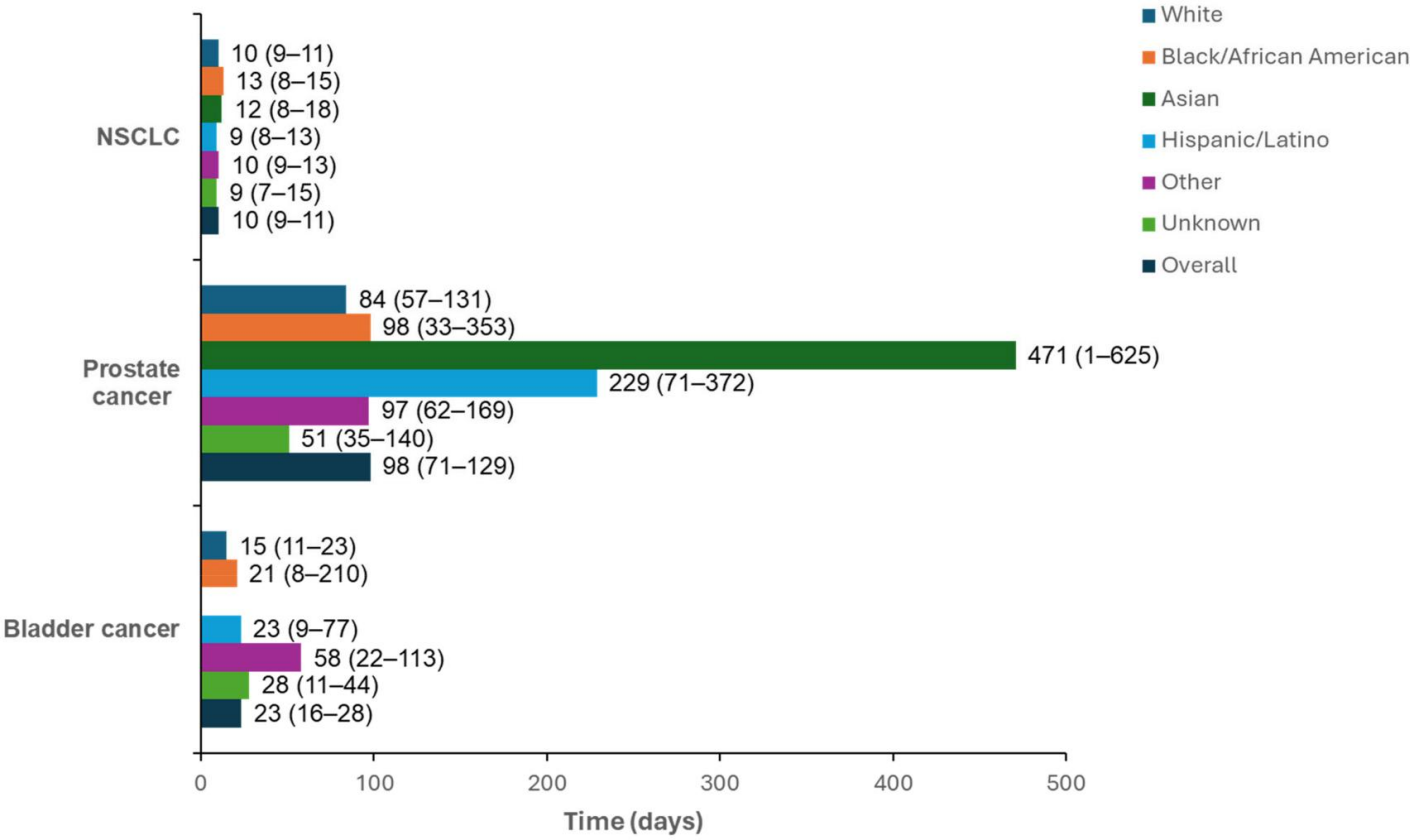
Time (days) from disease diagnosis to first biomarker test order by race and ethnicity in patients with metastatic NSCLC, prostate cancer, or bladder cancer. Data are presented as KM-estimated median (95% CI). CI, confidence interval; KM, Kaplan–Meier; NSCLC, non-small cell lung cancer.

In patients with metastatic disease, the KM-estimated median (95% CI) time from disease diagnosis to the first biomarker test order was 10 (9-11) days for NSCLC, 98 (71-129) days for prostate cancer, and 23 (16-28) days for bladder cancer (Figure 3). Variations in time from disease diagnosis to first biomarker test order were observed by patient-reported race and ethnicity (Figure 3). Estimates by race and ethnicity should be interpreted with caution due to small sample sizes in some subgroups.

### Actionable mutations in metastatic cancers

Actionable mutations indicated for targeted therapy for the three cancer types were identified from respective NCCN Guidelines at the time of the analysis (Table S1). Among patients who underwent biomarker testing, actionable mutations were detected in 29% (1729/5922) of those with NSCLC, 25% (418/1696) of those with prostate cancer, and 14% (53/371) of those with bladder cancer (Figure 1).

### Treatment initiation in metastatic disease

The proportion of patients initiating treatment prior to receipt of biomarker test results varied across tumor types. Of patients with metastatic NSCLC who had time-of-treatment records available, 23% (1097/4793) initiated treatment while test results were pending (Figure 1). In contrast, treatment was initiated before receipt of test results in 81% (1059/1315) of those with metastatic prostate cancer and 54% (135/252) of those with metastatic bladder cancer.

**Figure 4.**
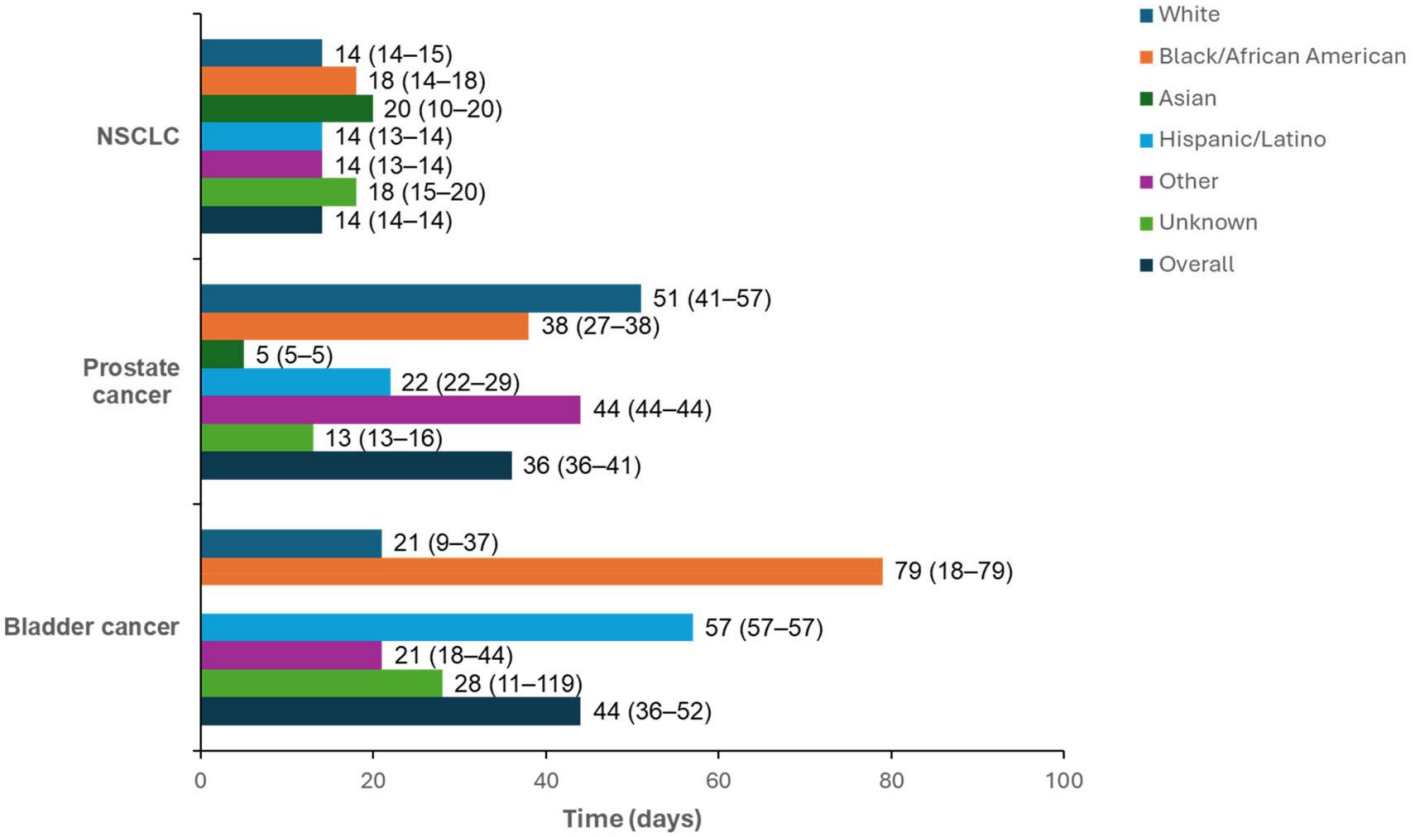
Time (days) from first biomarker test order to treatment initiation, according to race or ethnicity, in patients with metastatic NSCLC, prostate cancer, or bladder cancer. Data are presented as KM-estimated median (95% CI). CI, confidence interval; KM, Kaplan–Meier; NSCLC, non-small cell lung cancer.

Among patients with metastatic disease who had time-of-treatment records available, the KM-estimated median (95% CI) time from first biomarker test order to treatment initiation was 14 (14-14) days in patients with NSCLC, compared with 36 (36-41) days for those with prostate cancer and 44 (36-52) days for those with bladder cancer (Figure 4). Time to treatment initiation varied by race and ethnicity (Figure 4).

### Treatment received for metastatic disease

Among treated patients, the most common regimens for metastatic NSCLC were immunotherapy + chemotherapy (44% [3220/7383]) and immunotherapy (23% [1663/7383]). For metastatic prostate cancer, hormone therapy (71% [3551/4985]) was the most common treatment. Immunotherapy (43% [383/888]) and chemotherapy (38% [335/888]) were the most common treatment regimens for metastatic bladder cancer.

**Figure 5.**
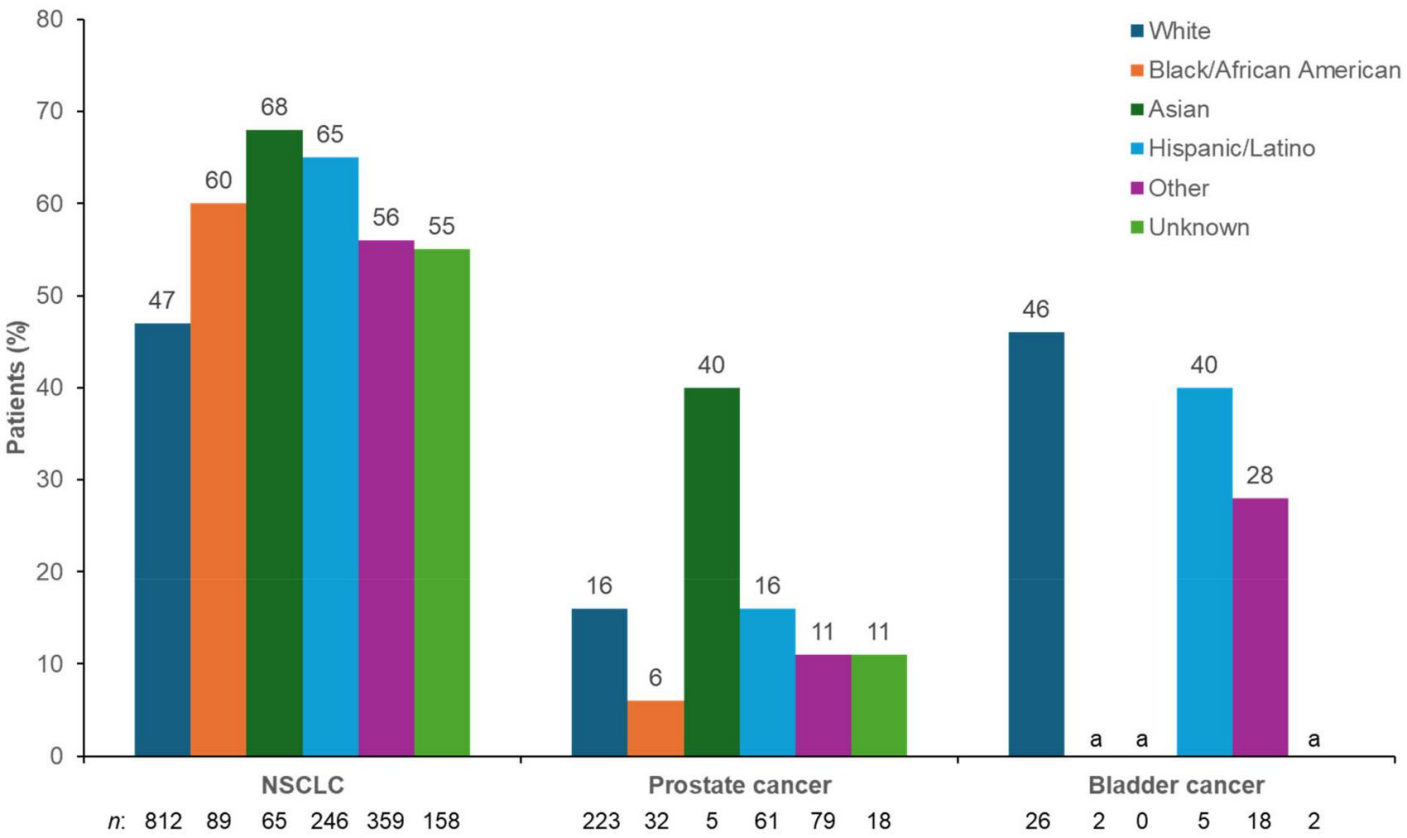
Proportion of patients with metastatic NSCLC, prostate cancer, or bladder cancer who had positive biomarker test results for actionable mutations and received targeted therapy, according to race or ethnicity. ^a^The numbers of patients testing positive were too low to provide reliable estimates. NSCLC, non-small cell lung cancer.

Targeted therapy was received by 13% (927/7383) of patients with metastatic NSCLC. However, use of targeted treatment remained low among those with metastatic prostate cancer (1% [61/4985]) or bladder cancer (2% [20/888]). Importantly, among patients with metastatic cancer and a proven actionable mutation, 54% (927/1729) with NSCLC, 15% (61/418) with prostate cancer, and 38% (20/53) with bladder cancer received targeted therapy (Figure 1). Among patients with actionable alterations, the KM-estimated median (95% CI) time from diagnosis to initiation of targeted therapy was 38 (35-41) days for NSCLC, 581 (518-659) days for prostate cancer, and 308 (200-443) days for bladder cancer. There were differences in the rates of targeted therapy according to race and ethnicity, but there was little difference according to primary healthcare insurance type (ie, commercial vs. Medicare/Medicaid) for any of the three cancer cohorts (Figure 5; Table S3).

To evaluate potential reasons for non-receipt of targeted therapy among patients with actionable alterations, the association of Eastern Cooperative Oncology Group performance status (ECOG PS) and insurance type with treatment use was examined. For each tumor type, chi-square tests were performed to compare receipt of targeted therapy across ECOG PS categories (0-1, 2, ≥3, unknown) and insurance groups (private, Medicare, Medicaid, other). Across tumor types, ECOG PS and insurance type were not significantly associated with receipt of targeted therapy. In NSCLC, both ECOG PS (*P* = .08) and insurance type (*P* = .05) demonstrated borderline trends toward association; however, no significant relationships were observed in prostate (ECOG PS, *P* = .38; insurance, *P* = .72) or bladder (ECOG PS, *P* = .84; insurance, *P* = .97) cancer.

Among patients with metastatic cancer who initiated therapy prior to receiving biomarker test results, 72% (248/346) with NSCLC, 12% (28/241) with prostate cancer, and 30% (7/23) with bladder cancer subsequently switched to targeted therapy after receiving a positive test result for an actionable mutation (Figure 1).

### Biomarker testing patterns over time: metastatic disease

**Figure 6.**
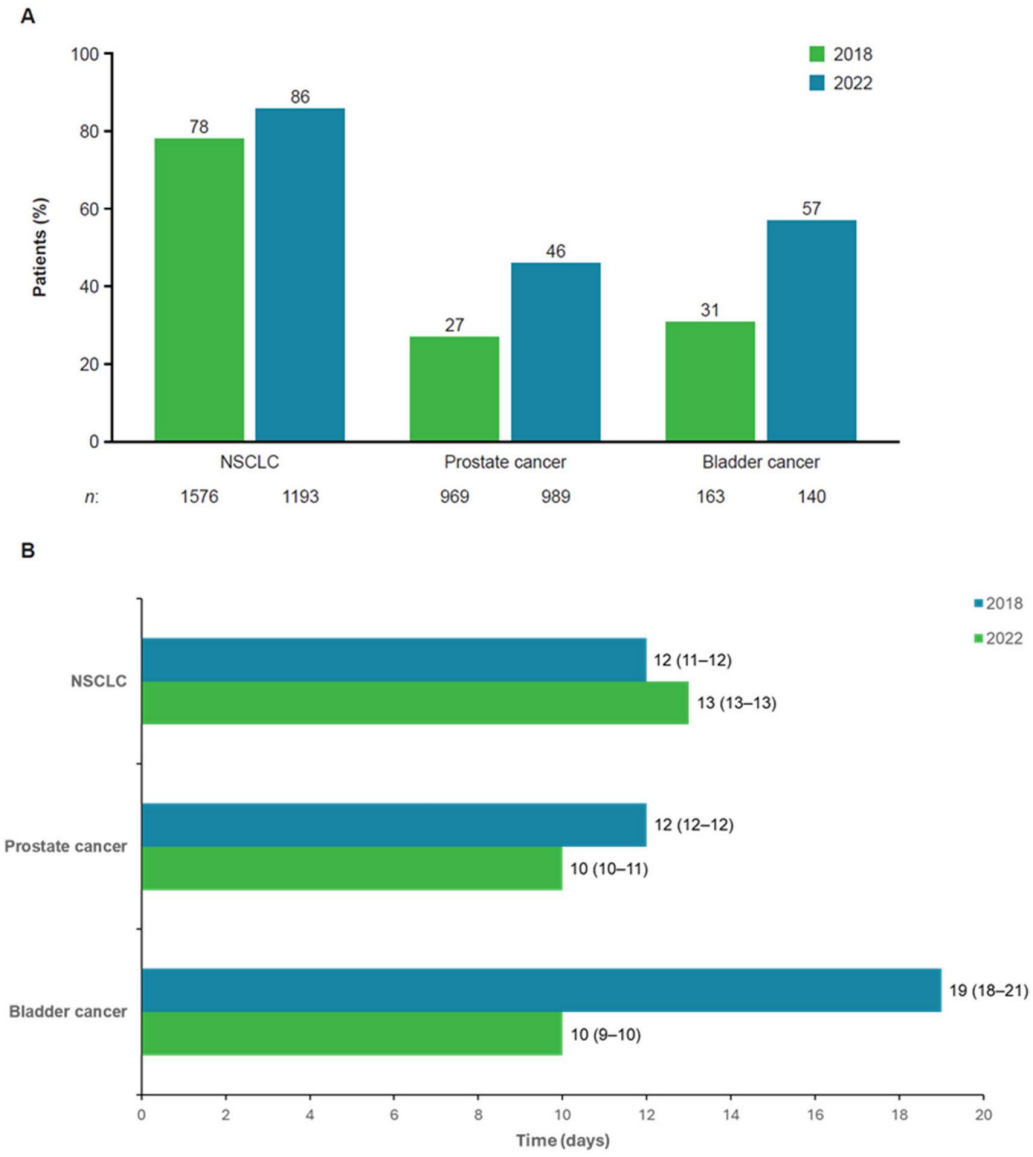
(A) Biomarker testing (total number of patients receiving biomarker tests during the full study period) and (B) KM-estimated median (95% CI) time (days) from test order to receipt of results among patients with metastatic NSCLC, prostate cancer, or bladder cancer in 2018 and 2022. CI, confidence interval; KM, Kaplan–Meier; NSCLC, non-small cell lung cancer.

The incidence of biomarker testing increased between 2018 and 2022 for patients in the three metastatic subgroups (Figure 6A). Among patients with metastatic NSCLC, 78% (1223/1576) underwent testing in 2018 versus 86% (1020/1193) in 2022. Those with metastatic prostate cancer had a notable increase in testing rate over time—from 27% (257/969) in 2018 to 46% (454/989) in 2022. Of those with metastatic bladder cancer, 31% (50/163) were tested in 2018 versus 57% (80/140) in 2022.

As the testing rates increased between 2018 and 2022, the KM-estimated median (95% CI) turnaround time from ordering a biomarker test to receipt of results was reduced for patients with metastatic prostate or bladder cancer (Figure 6B), with the greatest improvement for bladder cancer (from 19 [18-21] to 10 [9-10] days); for prostate cancer, turnaround time reduced from 12 (12-12) to 10 (10-11) days. In metastatic NSCLC, turnaround time was similar in 2018 and 2022 (12 [11-12] and 13 [13-13] days, respectively). Overall, biomarker testing rates increased and turnaround times decreased between 2018 and 2022. Key metrics are summarized by tumor type in Tables S4-S6.

## Discussion

The results of this analysis suggest that molecular biomarker testing rates in NSCLC have improved in community settings in the United States, whereas testing for prostate and bladder cancer remains lower and more variable, particularly for patients with advanced-stage/metastatic disease. While the percentage of patients tested increased and turnaround times for test results improved between 2018 and 2022, roughly half of newly diagnosed patients with metastatic prostate or bladder cancer received biomarker testing. Molecular testing patterns and time to treatment initiation varied by cancer type, with higher testing rates and shorter turnaround times for metastatic NSCLC compared with metastatic prostate and bladder cancers. Disparities in biomarker testing rates, turnaround times, and the use of targeted treatment were observed among certain racial and ethnic groups. These findings should be interpreted in the context of a large regional community oncology network and may not fully reflect national practice patterns.

The discovery of actionable mutations and the development of targeted therapies have revolutionized NSCLC treatment, and biomarker-directed therapy in NSCLC is associated with improved outcomes.^26^ A recent analysis of the Surveillance, Epidemiology, and End Results (SEER) program reported a biomarker test rate of 39% in NSCLC;^6^ however, the rate in the current study was 63% in the overall NSCLC cohort. Reported biomarker testing rates in metastatic NSCLC vary widely across real-world studies, with recent studies suggesting rates approaching 80%-90% in advanced disease, particularly when defined as receipt of any biomarker testing.^27^^,^^28^ Lower estimates, such as those observed in the SEER-based analyses, may reflect earlier study periods, inclusion of mixed-stage populations, or incomplete capture of internal testing. Recent studies show higher rates of biomarker testing in the community oncology setting among patients with advanced NSCLC and solely non-squamous metastatic disease.^5^^,^^7^^,^^29^ Consistent with this variability, the current study observed higher testing rates among those with metastatic disease at diagnosis (80%). In contrast to NSCLC, where biomarker testing has become increasingly standardized due to a broader range of actionable alterations and established treatment pathways, testing in prostate cancer and bladder cancer remains more heterogeneous and continues to evolve, with reported rates of 38%-59% and 45%, respectively.^9^^,^^30^^,^^31^

In prostate cancer, expert consensus opinion and NCCN Guidelines recommend multigene germline and somatic genetic testing in metastatic or high-risk/very high-risk localized disease to identify potential actionable mutations in HRR genes and inform treatment with poly(ADP-ribose) polymerase (PARP) inhibitors.^2^^,^^32^^,^^33^ Biomarker testing rates for metastatic prostate cancer remained low (34%) within this community oncology practice setting (although an increasing trend is observed between 2018 and 2022) despite approvals for PARP inhibitors during this period. An analysis of EMR data from 5213 patients with metastatic castration-resistant prostate cancer (CRPC) obtained between 2013 and 2019 reported similar findings; only 13% of patients completed genetic testing for HRR genes.^13^ Another study found that next-generation sequencing (NGS) was performed infrequently in patients with prostate cancer (ie, at approximately one-tenth of the rate of that in patients with lung cancer).^34^ Higher HRR test rates have been reported among patients with metastatic disease: 29% of patients had NGS testing in one analysis of 11 927 patients with metastatic prostate cancer treated at both community and hospital-based settings,^10^ while 38% of 9395 patients with metastatic CRPC largely treated in the community setting had biomarker testing.^9^ This variability likely reflects differences in disease-state–specific guideline recommendations (eg, metastatic CRPC vs. hormone-sensitive disease), evolving indications for biomarker-directed therapies, and logistical challenges in integrating germline and somatic testing into routine workflows.

NCCN Guidelines for bladder cancer specify that testing for patients with locally advanced/metastatic disease should include analysis for *FGFR3* genetic alterations based on the US Food and Drug Administration approval for erdafitinib.^4^ A study among 6490 patients with advanced urothelial cancer indicated that only 32% received NGS testing.^10^ Estimates from the current study for patients with metastatic bladder cancer show an increase in testing rate from 31% in 2018 to 57% in 2022.

The time from cancer diagnosis to first biomarker test order and from first test order to treatment initiation was markedly shorter for patients with metastatic NSCLC versus bladder cancer or prostate cancer in this study. The study also detected differences in molecular testing patterns by race and ethnicity, although these results should be interpreted with caution due to small sample sizes in some subgroups. Racial, ethnic, and socioeconomic differences in biomarker testing have been noted in previous reports.^6^^,^^10^ The SEER program found that for NSCLC, including metastatic disease, Black/African American patients and those from reduced socioeconomic areas were significantly less likely to undergo biomarker testing versus White patients and those from higher socioeconomic areas.^6^ A retrospective analysis of a large US EMR database of patients with metastatic prostate cancer and advanced bladder cancer also found that Black/African American patients had lower rates of molecular testing than White patients, as did those with low versus high socioeconomic status and Medicare/Medicaid versus other insurance.^10^ However, an analysis of the PROMISE registry found no difference in biomarker testing between Black/African American and White men with metastatic CRPC.^35^

Addressing barriers to molecular testing among minoritized groups may help reduce disparities in cancer care. Black/African American patients are twice as likely to die from prostate cancer^36^ and more likely to be diagnosed with advanced bladder cancer^37^ versus White patients. Barriers to molecular biomarker testing include limited patient awareness and comprehension of the importance of testing and the process, distrust of the healthcare system, lack of access to genetic counseling, costs, insurance coverage, billing practices, tissue adequacy, and clinical capacity; these barriers can be particularly relevant among minoritized and rural populations.^32^^,^^38–40^

Socioeconomic disparities in biomarker testing access and outcomes in cancer are well known. A retrospective analysis of a large US healthcare database showed that Medicaid beneficiaries were 19% less likely to receive biomarker testing and 30% less likely to receive first-line biomarker-driven therapy than commercially insured patients, possibly contributing to a higher observed risk of death.^15^ However, no major differences were found in targeted treatment rates between patients with commercial versus Medicare/Medicaid coverage, regardless of cancer type.

In this study, treatment was withheld until biomarker test results were obtained for most patients with metastatic NSCLC, consistent with previous reports.^7^ In contrast, treatment was initiated before test results were known for most patients with metastatic prostate cancer and approximately half of patients with metastatic bladder cancer, which may represent a missed opportunity to receive the most effective treatment in the frontline setting in some cases. However, initiation of treatment prior to receipt of biomarker results may also reflect appropriate clinical decision-making in the setting of symptomatic disease burden, rapidly progressive disease, and the need to avoid delays in care. Overall, only 12% of patients with metastatic prostate cancer whose treatment was initiated prior to obtaining biomarker test results were changed to targeted therapy, even when actionable mutations were identified. On the other hand, approximately 72% of patients with metastatic NSCLC whose treatment was initiated prior to obtaining biomarker test results were changed to targeted therapy upon receipt of a positive test for an actionable mutation (Figure 1).

In a previous study using EMR data from 1260 patients with advanced NSCLC, 48% with actionable mutations received targeted therapy.^26^ Similarly, in the current study, targeted therapy use was suboptimal across tumor types. ECOG PS and insurance type were not significantly associated with receipt of targeted therapy among patients with actionable alterations, suggesting that neither clinical fitness nor access-related factors sufficiently explain the observed underuse of targeted therapy. Instead, the gap between identification of actionable alterations and receipt of targeted therapy may reflect other unmeasured clinical or system-level factors not captured in structured data, including rapid clinical decline, comorbidities, payer-related barriers, and patient preference.

These data support the need for a deeper evaluation of the barriers to biomarker testing for all eligible patients with prostate or bladder cancer and the need to address both educational and system-level barriers among community practitioners. These barriers may include workflow inefficiencies, delays in test ordering and processing, tissue adequacy, and payer-related constraints, in addition to gaps in testing awareness/requirements or familiarity with evolving guidelines. Potential strategies to address these gaps include implementation of reflex biomarker testing protocols, standardized testing pathways for eligible patients, and enhanced care coordination to monitor testing turnaround times and facilitate timely treatment decisions. The American Cancer Society National Lung Cancer Roundtable recently advocated for comprehensive biomarker testing for all eligible patients with NSCLC through the development of educational materials and clinical decision tools.^41^ Similar initiatives are needed to assist decision-making for biomarker testing in prostate and bladder cancers.

### Limitations

Although this study has limitations inherent to retrospective EMR analyses, it also has some strengths. The analysis evaluated real-world biomarker testing patterns, turnaround times, and targeted therapy use over multiple tumor types within a large regional community oncology network. In contrast to prior studies focused on a single malignancy or limited time period, this analysis examined longitudinal trends across NSCLC, prostate cancer, and bladder cancer between 2018 and 2022, enabling assessment of evolving testing practices. The inclusion of treatment sequencing relative to biomarker testing and evaluation of racial and ethnic differences further provides relevant context regarding potential gaps in access and care delivery.

Several limitations should be acknowledged. First, clinical factors influencing biomarker testing and treatment decisions, such as symptom burden, disease acuity (eg, visceral crisis), patient preference, and contraindications, were not consistently captured in structured fields and could not be evaluated. Second, line-of-therapy information and disease-state stratification (eg, metastatic hormone-sensitive prostate cancer vs. CRPC) were not available, limiting interpretation of treatment patterns and targeted therapy uptake. Third, biomarker definitions and actionability were applied uniformly across the study period and may not fully reflect evolving guidelines, regulatory approvals, and changes in standard of care between 2018 and 2022. In addition, information on test failure or insufficient tissue rates was incomplete, limiting assessment of pre-analytic factors influencing turnaround time. As with analyses of real-world data, capture of biomarker testing depends on the completeness of documentation in the EMR and integration of external laboratory results; incomplete capture may have resulted in underestimation of testing rates. The resulting heterogeneity and missingness may also contribute to variability in observed outcomes. Another limitation is that the study population may not be fully generalizable to other practice settings or national populations. The NSCLC stage distribution was broadly consistent with population-based epidemiology, whereas the prostate and bladder cancer cohorts appeared enriched for advanced-stage disease relative to SEER expectations, particularly due to higher proportions of stage IV and unknown-stage diagnoses. Finally, clinical outcomes such as progression-free survival or overall survival were not assessed, and patients with more complete testing records or smoother care coordination may have been overrepresented. All analyses were descriptive in nature; therefore, findings should be interpreted as hypothesis-generating rather than causal.

## Conclusions

In this analysis of a large regional community oncology network, biomarker testing rates and treatment patterns varied substantially across tumor types. Testing was highest in metastatic NSCLC and lower in metastatic prostate and bladder cancer, reflecting differences in guideline maturity, biomarker availability, and clinical integration during the study period. Although testing rates and turnaround times improved between 2018 and 2022, gaps remain in the alignment of biomarker identification with targeted therapy use, particularly in prostate and bladder cancer. These findings suggest opportunities to optimize testing workflows and reduce delays in the availability of results to better support treatment decision-making. Addressing these gaps will require a combination of educational and system-level interventions, including standardized testing pathways, improved care coordination, and strategies for timely access to biomarker results. Further research is needed to better understand the drivers of testing variability and to evaluate the relationship between biomarker testing, targeted therapy use, and clinical outcomes in real-world settings.

## Supplementary Material

oyag243_Supplementary_Data

## Data Availability

Data that support the findings of this study are not publicly available because of patient privacy and data licensing restrictions but may be available from the corresponding author on reasonable request and with permission of the data provider.
