# Supplementary material for: Biomarker testing patterns among patients newly diagnosed with metastatic non-small cell lung cancer, prostate cancer, and bladder cancer
Source: Oncologist. 2026 Jun 27;31(8):oyag243. doi: 10.1093/oncolo/oyag243 (PMC13348720; doi:10.1093/oncolo/oyag243)
Supplement: oyag243_Supplementary_Data [file oyag243_supplementary_data.zip › Biomarker Testing The Oncologist MS_1June2026_suppl.docx]

**Supplementary Table S1.** Tumor-specific biomarker testing definitions.

| **Cancer type** | **Actionable biomarker mutations** |
| --- | --- |
| NSCLC | *EGFR* (ex19del, ex21L858R, S768I, L861Q, G719X, ex20i)  *KRAS* G12C  *ALK* rearrangement  *ROS1* rearrangement  *BRAF* V600E  *NTRK1/2/3* gene fusion  *MET* ex14skip  *RET* rearrangement  *HER2* |
| Prostate cancer | *FGFR2*  *FGFR3* |
| Bladder cancer | *BRCA1*  *BRCA2*  *ATM*  *BARD1*  *BRIP1*  *CDK12*  *CHEK1*  *CHEK2*  *FANCL*  *PALB2*  *RAD51B*  *RAD51C*  *RAD51D*  *RAD54L* |

Includes any mutation in the indicated gene, unless otherwise noted.

**Supplementary Table S2.** Testing modality data for patients in the overall study population who received biomarker testing.

| **Parameter** | **NSCLC**  **(*n*=8094)** | **Prostate cancer**  **(*n*=1976)** | **Bladder cancer**  **(*n*=732)** |
| --- | --- | --- | --- |
| Type of testing  Multigene panel  Single-gene  Panel and single-gene  Unknown | 4325 (53)  2550 (32)  1171 (14)  48 (<1) | 1772 (90)  160 (8)  37 (2)  7 (<1) | 569 (78)  131 (18)  32 (4)  0 |
| Specimen type  Tissue  Plasma (liquid biopsy)  Tissue and plasma  Unknown | 6505 (80)  740 (9)  545 (7)  304 (4) | 719 (36)  884 (45)  174 (9)  199 (10) | 498 (68)  103 (14)  40 (5)  91 (12) |
| Test result data source  Discrete data  Scanned documents  Discrete data and scanned documents | 3751 (46)  4282 (53)  61 (<1) | 1193 (60)  776 (39)  7 (<1) | 398 (54)  309 (42)  25 (3) |
| Time (days) from test order to receipt of results, KM-estimated median (95% CI)  By testing type  Multigene panel  Single-gene  Unknown  By specimen type  Tissue  Plasma (liquid biopsy)  Unknown | 14 (14–14)  10 (10–10)  3 (2–4)  14 (14–14)  11 (11–11)  12 (12–12) | 12 (11–12)  8 (8–9)  7 (4–7)  14 (14–15)  11 (11–11)  11 (7–13) | 13 (13–13)  22 (19–22)  –  15 (14–16)  11 (11–11)  13 (11–19) |

Data are presented as *n* (%) unless otherwise stated.
Abbreviations: CI, confidence interval; KM, Kaplan–Meier; NSCLC, non-small cell lung cancer.

**Supplementary Table S3.** Proportion of patients with metastatic NSCLC, prostate cancer, or bladder cancer who had positive biomarker test results and received targeted therapy, according to race and ethnicity and primary healthcare insurance type.

| **Parameter** | **Metastatic NSCLC**  **(*n*=1729)** | **Metastatic prostate cancer**  **(*n*=418)** | **Metastatic bladder cancer**  **(*n*=53)** |
| --- | --- | --- | --- |
| All patients with metastatic disease  Race or ethnicity  White  Black/African American  Asian  Hispanic/Latino^a^  Other^b^  Unknown | 927/1729 (54)  384/812 (47)  53/89 (60)  44/65 (68)  159/246 (65)  200/359 (56)  87/158 (55) | 61/418 (15)  36/223 (16)  2/32 (6)  2/5 (40)  10/61 (16)  9/79 (11)  2/18 (11) | 20/53 (38)  12/26 (46)  0  0  2/5 (40)  5/18 (28)  1/2 (50) |
| Primary insurance type |  |  |  |
| Commercial | 590/1061 (56) | 38/250 (15) | 10/28 (36) |
| Medicare/Medicaid | 331/659 (50) | 23/166 (14) | 10/25 (40) |
| Unknown | 6/9 (67) | 0 | 0 |

Data are presented as *n*/*N* (%).
^a^Includes Hispanic or Latino American Indian/Alaska Native, Hispanic Asian, Hispanic Black or African American, Hispanic multirace, Hispanic Native Hawaiian/Other Pacific Islander, Hispanic other, and Hispanic White (but excludes unknown/declined to inform).
^b^Non-Hispanic American Indian/Alaska Native, Non-Hispanic multirace, Non-Hispanic Native Hawaiian/Other Pacific Islander, and Non-Hispanic Other. 
NSCLC, non-small cell lung cancer.

**Supplementary Table S4.** Key metrics by year (2018 vs 2022) for NSCLC.

| **Parameter** | **2018** | **2022** |
| --- | --- | --- |
| Patients who received biomarker testing  Overall  Stage IV | 2389/3805 (63)  1223/1576 (78) | 2139/3260 (66)  1020/1193 (85) |
| Time (days) from biomarker test order to receipt of results, KM-estimated median (95% CI)  Stage IV | 12 (11–12) | 13 (13–13) |
| Patients who initiated treatment prior to receipt of test results  Overall | 421/1641 (26) | 341/1484 (23) |
| Patients who tested positive for an actionable mutation and received targeted therapy  Overall | 245/531 (46) | 243/530 (46) |

Data are presented as *n*/*N* (%) unless otherwise stated.

CI, confidence interval; KM, Kaplan–Meier; NSCLC, non-small cell lung cancer.

**Supplementary Table S5.** Key metrics by year (2018 vs 2022) for prostate cancer.

| **Parameter** | **2018** | **2022** |
| --- | --- | --- |
| Patients who received biomarker testing  Overall  Stage IV | 581/3831 (15)  257/969 (27) | 811/4184 (19)  454/989 (46) |
| Time (days) from biomarker test order to receipt of results, KM-estimated median (95% CI)  Stage IV | 12 (12–12) | 10 (10–11) |
| Patients who initiated treatment prior to receipt of test results  Overall | 239/281 (85) | 313/438 (71) |
| Patients who tested positive for an actionable mutation and received targeted therapy  Overall | 13/96 (14) | 7/191 (4) |

Data are presented as *n*/*N* (%) unless otherwise stated.

CI, confidence interval; KM, Kaplan–Meier.

**Supplementary Table S6.** Key metrics by year (2018 vs 2022) for bladder cancer.

| **Parameter** | **2018** | **2022** |
| --- | --- | --- |
| Patients who received biomarker testing  Overall  Stage IV | 164/724 (23)  50/163 (31) | 297/840 (35)  80/140 (57) |
| Time (days) from biomarker test order to receipt of results, KM-estimated median (95% CI)  Stage IV | 19 (18–21) | 10 (9–10) |
| Patients who initiated treatment prior to receipt of test results  Overall | 50/80 (63) | 103/165 (62) |
| Patients who tested positive for an actionable mutation and received targeted therapy  Overall | 8/16 (50) | 6/37 (16) |

Data are presented as *n*/*N* (%) unless otherwise stated.

CI, confidence interval; KM, Kaplan–Meier.
